# Comparative [18F]FDG and [18F]DPA714 PET imaging and time-dependent changes of brown adipose tissue in tumor-bearing mice

**DOI:** 10.1080/21623945.2020.1814546

**Published:** 2020-09-09

**Authors:** Na Niu, Haiqun Xing, Xuezhu Wang, Jie Ding, Zhixin Hao, Chao Ren, Jiantao Ba, Lianfang Zheng, Chao Fu, Haiyan Zhao, Li Huo

**Affiliations:** Department of Nuclear Medicine, Peking Union Medical College Hospital, Chinese Academy of Medical Sciences and Peking Union Medical College (CAMS & PUMC), Beijing, China

**Keywords:** Brown adipose tissue, [^18^F]DPA714, [^18^F]FDG, PET, tumour, energy metabolism

## Abstract

Brown adipose tissue (BAT) is important in monitoring energy homeostasis and cancer cachexia. Different from white adipose tissue, BAT is characterized by the presence of a large number of mitochondria in adipocytes. Translocator protein 18 kDa (TSPO), a critical transporter, is expressed in the outer membrane of mitochondria. We speculated that [^18^F]DPA714, a specific TSPO tracer, may monitor BAT activity in tumor-bearing mice in vivo. We first analyzed the radioactive uptake of positron emission tomography (PET) tracers in BAT of CT26 xenograft mice with 18F-fluorodeoxyglucose ([^18^F]FDG) and [^18^F]DPA714. We also studied the BAT uptake of [^18^F]DPA714 in CT26, A549 and LLC tumor models. The dynamic distribution of [^18^F]FDG is quite variable among animals, even in mice of the same tumor model (%ID/g-mean: mean ± SDM, 8.61 ± 8.90, n = 16). Contrarily, [^18^F]DPA714 produced high-quality and stable BAT imaging in different tumor models and different animals of the same model. Interestingly, %ID/g-mean of [^18^F]DPA714 in BAT was significantly higher on day 26 than that on day 7 in CT26 xenograft model. Taken together, these results strongly indicate the potential feasibility of [^18^F]DPA714 PET imaging in investigating BAT and energy metabolism during tumor progression in preclinical and clinical study.

## Introduction

Brown adipose tissue (BAT) plays a key role in non-shivering thermogenesis, glucose and lipid metabolism, insulin resistance and endogenous energy consumption in mammals [1–3]. Numerous studies indicate that activation of BAT can counter obesity and ameliorate metabolic diseases [4,5]. However, the activation of BAT is harmful to the patients with malignant tumour. Accumulated studies suggest that almost all malignant tumours share the phenomena of continuous transforming of white adipose tissue (WAT) into BAT, which will cause irreversible cancer cachexia [6]. Scholars have found that the inflammatory factor interleukin-6 (IL-6) and parathyroid hormone related proteins (PTHrP), secreted by tumour tissues, are closely relevant to the adipose tissue browning process [7,8]. Furthermore, anti-inflammatory drugs can avoid browning of fat tissue and cancer cachexia [9]. Therefore, it is of great clinical value to monitor the activity and changes of BAT by effective, non-invasive and *in vivo* imaging methods.

Several molecular imaging methods have been used for BAT imaging. At present, ^18^F-fluorodeoxyglucose ([^18^F]FDG) positron emission tomography (PET) is the main approach to monitor BAT activity, and it is based on the high expression of glucose transporter in BAT [10]. However, the imaging results of [^18^F]FDG PET are quite discrepant and vulnerable to environmental influences [11]. In the retrospective studies, the prevalence or positive rate of BAT in measurement was less than 5% [12,13]. ^99m^Tc-methoyl isobutyl isonitrile (MIBI) and ^123^I-meta-iodobenzylguanidine (MIBG) single-photon emission computed tomography (SPECT) can also be used in BAT imaging, but the low positive rate limits their application [14]. Conventional magnetic resonance imaging (MRI) scan can also identify WAT and BAT, the principle of which is that BAT has less fat and more water [15]. But, MRI is lack of specificity and its value is also limited. PET/MR and other multimodal imaging methods will play a greater and dominant role in the study of BAT activity.

Translocator protein 18 kDa (TSPO), expressed in the outer membrane of mitochondria, is a reliable inflammatory biomarker and related to many human diseases, including metastatic cancer, inflammatory and neurological diseases (such as Alzheimer’s disease and Parkinson’s disease) [16–18]. An important feature of BAT is the large quantity of mitochondria in the cytoplasm. Previous studies have proven the higher radioactive uptake in BAT by specific TSPO-based PET tracers, including ^11^C-PBR28 and [^18^F]DPA [19,20]. [^18^F]DPA714, which is derived from the nature ligand of TSPO and has been used in clinical trials, is a highly TSPO specific and practically mature PET tracer [21]. However, direct comparison of [^18^F]FDG and [^18^F]DPA714 as PET tracers in the imaging of BAT has not been reported.

In this study, we first compared the PET imaging effects and applicability of [^18^F]FDG and [^18^F]DPA714 in BAT of the same tumour model. Then, we investigated the performance of [^18^F]DPA714 in the BAT of different tumour-bearing mice that may result in cachexia. We also studied the uptake of [^18^F]DPA714 in BAT at different stages of the tumour model.

## Materials and methods

### Animals

Nude mice were obtained from Beijing Vital River Laboratory Animal Technology Co., Ltd (Beijing, China). All mice were kept in a biologically clean environment under 12 h light/dark cycles and with controlled temperature of (23 ± 1) °C and relative humidity of 50%-70%, and provided with a standard rodent chow diet and water *ad libitum*. All animal experiments were conducted in compliance with the Guide for the Care and Use of Medical Laboratory Animals (Ministry of Health, China) and institutional guidelines of Beijing Union Hospital.

### Tumour modelling cells

Human lung cancer cells (A549), mice colorectal cancer cells (CT26) and mice lung cancer cells (LLC) were purchased from American Type Culture Collection (ATCC). These cells were cultured in a cell medium containing 10% foetal bovine serum. One portion of 1 × 10^6^ cells was injected in the left limb of each animal to induce a corresponding tumour model of mice.

### Radiochemistry

The commercial ^18^F ion was produced by SUMITOMO cyclotron (HM-7, Sumitomo Heavy Industry, Tokyo, Japan). Radio-synthesis of [^18^F]FDG was performed as classic studies [22]. The radiolabeling process of [^18^F]DPA714 was similar to the reported protocol in literature [23]. Simply, ^18^F ion was first captured by QMA column (Sep-Pak® Light QMA, Waters Corporation, Milford, Massachusetts USA), then eluted into the reaction tube with K22/K_2_CO_3_ solution, and added with 2 mL of anhydrous acetonitrile. Following azeotropic water removal, the reaction system was cooled to room temperature after completion. Next, add 1 mL of DPA714 precursor dissolved in anhydrous acetonitrile to the reaction tube, and raise the temperature to 100°C for fluorination reaction for 10 min, and cool the system to room temperature after reaction. Then, add 10 mL of water to dilute the reaction system, inject the diluted solution to C18-column for separation, wash the column with 20 mL of water, and elute the labelled product with 1 mL of anhydrous ethanol. Take a portion of the radiolabeled product for identification and preparation purity assay by preparative HPLC, with [^19^F]DPA714 as the standard reference, and collect the target peak. The final radiochemical purities of products [^18^F]FDG and [^18^F]DPA714 were both >95%.

### MicroPET imaging and data processing

All tumour-bearing mice were scanned with microPET (Inveon, Siemens). During the scan, the mice were anesthetized continuously by inhalation of 2% isoflurane. All animals were given (100 ± 10) μCi of radiolabeled tracer by tail vein injection. Static whole-body microPET scans (10 min per mouse) for [^18^F]FDG were conducted at 45 min after injection, and the microPET scans (15 min per mouse) were performed at 60 min (or 30 min for distribution analysis) after [^18^F]DPA714 administration. After scanning, data reconstruction (including reconstruction algorithm, number of iterations, and type of filter after reconstruction) was handled by the Inveon Acquisition Workplace (IAW) software (Siemens Inveon). The regions of interest (ROIs) were delineated in the reconstructed images by manual selection of the statistical investigator who was blind to experimental design and grouping. PMOD software (PMOD Technologies Ltd) was used for analysing the data of radioactive uptake (%ID/g) in the ROIs after reconstruction and quantification.

### Statistical analysis

All statistical analysis was performed by SPSS 19.0 software (SPSS Inc., Chicago, Illinois, USA). Quantitative data are expressed as ‘means ± SDM’, and comparison of quantitative data between two groups was analysed by unpaired t-test.

## Results

### [^18^F]FDG and [^18^F]DPA714 PET imaging of BAT in CT26 xenograft mice

**Figure 1.**
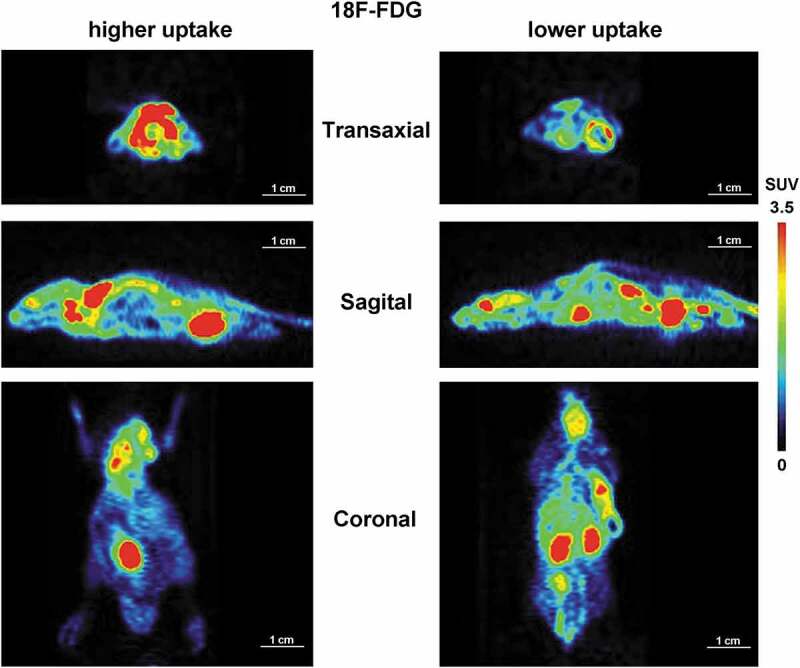
Static whole-body microPET imaging of CT26 tumour-bearing mice with [^18^F]FDG

[^18^F]FDG PET imaging is a classic imaging method to detect the BAT activity. In this research, taking [^18^F]FDG imaging as control, we comparatively studied the differences between [^18^F]DPA714 and [^18^F]FDG PET imaging for the monitoring of BAT activity in the same tumour-bearing mouse model. When the tumour volumes of CT26 model increased to about 500 mm^3^, [^18^F]FDG scanning was performed on the first day and [^18^F]DPA714 scanning was performed on the third day. As shown in Figure 1, the radioactive uptake of [^18^F]FDG in BAT of the scapular region in the CT26 tumour-bearing animals varied dramatically. Of the sixteen mice scanned (%ID/g-mean = 8.61 ± 8.90, n = 16), seven had significant BAT uptake in the scapular region (%ID/g-mean = 15.84 ± 9.35, n = 7), while the BAT uptake of [^18^F]FDG in the other nine animals was not obvious (%ID/g-mean = 2.99 ± 1.31, n = 9). Moreover, in accordance with previous studies, our PET imaging showed that [^18^F]FDG was mainly distributed in the heart, liver, brain and tumour. The data summarized from the results of CT26 tumour uptake of [^18^F]FDG are shown in S Table 1 in the Supplemental Information.

The representative images (at transaxial, sagittal and coronal sections) showing the radioactive uptake of [^18^F]FDG in whole body of the CT26 tumour-bearing mice. The [^18^F]FDG uptake in the BAT of the scapular region in this tumour model was distinguished between animals. Among the 16 mice, 7 had significant BAT uptake in the scapular region (as shown in the left, ‘higher uptake’, %ID/g-mean = 15.84 ± 9.35, *n* = 7), while the other 9 animals had no obvious BAT [^18^F]FDG uptake in the scapular region (shown in the right, ‘lower uptake’, %ID/g-mean = 2.99 ± 1.31, *n* = 9). The PET imaging also showed obvious [^18^F]FDG uptake in the heart, liver, brain and tumour in the CT26 tumour models. SUV, Standard Uptake Value of the radioactivity.

**Figure 2.**
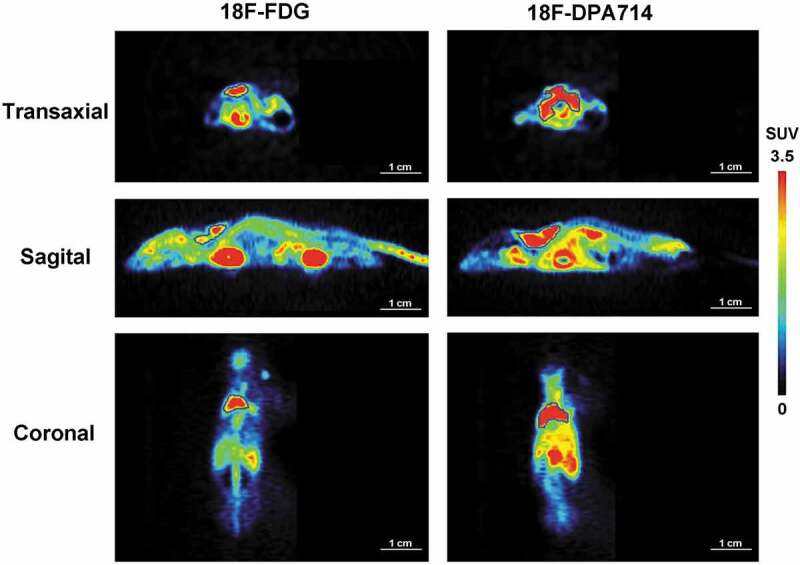
Characteristics of biodistribution of [^18^F]FDG and [^18^F]DPA714 in the CT26 tumour model

Interestingly, as shown in Figure 2, the biodistribution characteristics of [^18^F]DPA714 in mice were significantly different from that of [^18^F]FDG. In the same CT26 xenograft model mice, the BAT uptake of [^18^F]DPA714 (%ID/g-mean = 15.72 ± 3.32, n = 6) in the scapular region was significantly higher than that of [^18^F]FDG (%ID/g-mean = 6.14 ± 4.03, n = 6; p < 0.01 vs. [^18^F]DPA714). The uptake values of [^18^F]DPA714 (%ID/g-mean) at different time points in six organs (including tumour) in CT26 tumour-bearing mice were calculated and presented in supplemental S Figure 1. Compared with most of the other major organs, the uptake of [^18^F]DPA714 in tumour tissue was relatively lower at all the time points.

The biodistribution of [^18^F]DPA714 in the CT26 tumour-bearing mice was significantly different from that of [^18^F]FDG. The BAT uptake of [^18^F]DPA714 in the scapular region was significantly higher than that of [^18^F]FDG (*p* < 0.01). [^18^F]DPA714 uptake in the scapular region was similar and consistent between all the scanned tumour-bearing mice. *n* = 6 mice for each group. Delineated ROIs for the scapular BAT regions are shown in the images.

### [^18^F]DPA714 uptake in BAT of different tumour-bearing models

**Figure 3.**
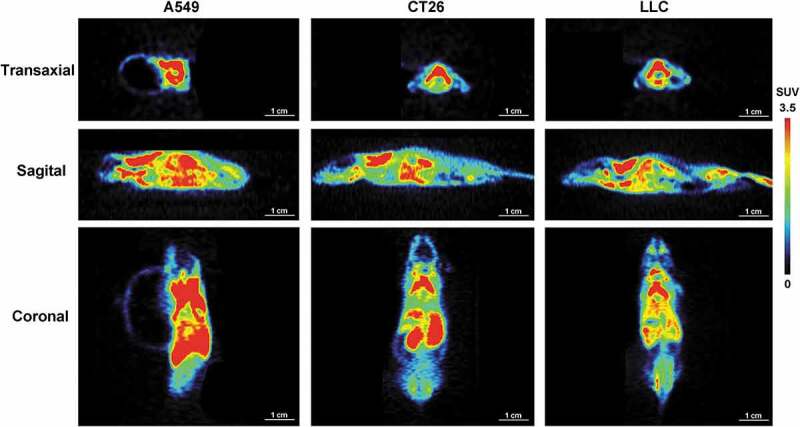
Biodistribution and BAT uptake of [^18^F]DPA714 in different tumour models

Then, in order to study the stability and durability in application of the tracer in BAT imaging, we investigated the variation of the uptake of [^18^F]DPA714 in different kinds of tumour-bearing models. Considering the representativeness and differences of species and location, we chose CT26, A549 and LLC tumour models (n = 6 mice for each model group) for the study of the usability characteristics of [^18^F]DPA714 as a PET tracer for BAT monitoring. The average tumour volume of each group was (500 ± 100) mm^3^ at the scanning time point. As shown in Figure 3, the radioactive uptake of [^18^F]DPA714 in the scapular region was all relatively high in the three tumour-bearing models (CT26, %ID/g-mean = 15.72 ± 3.32, n = 6; A549, %ID/g-mean = 21.16 ± 7.49, n = 6; and LLC, %ID/g-mean = 22.48 ± 8.22, n = 5).

A549 (*n* = 6 mice), CT26 (*n* = 6) and LLC (*n* = 5) tumour models were used for the study of the characteristics of [^18^F]DPA714 as an imaging tracer for BAT monitoring. Average tumour volume was (500 ± 100) mm^3^ for each group at microPET scanning. Radioactive uptake of [^18^F]DPA714 in the scapular region of the different tumour models was all relatively high and consistent with each other group.

### Longitudinal [^18^F]DPA714 PET imaging of BAT in CT26 xenograft mice

**Figure 4.**
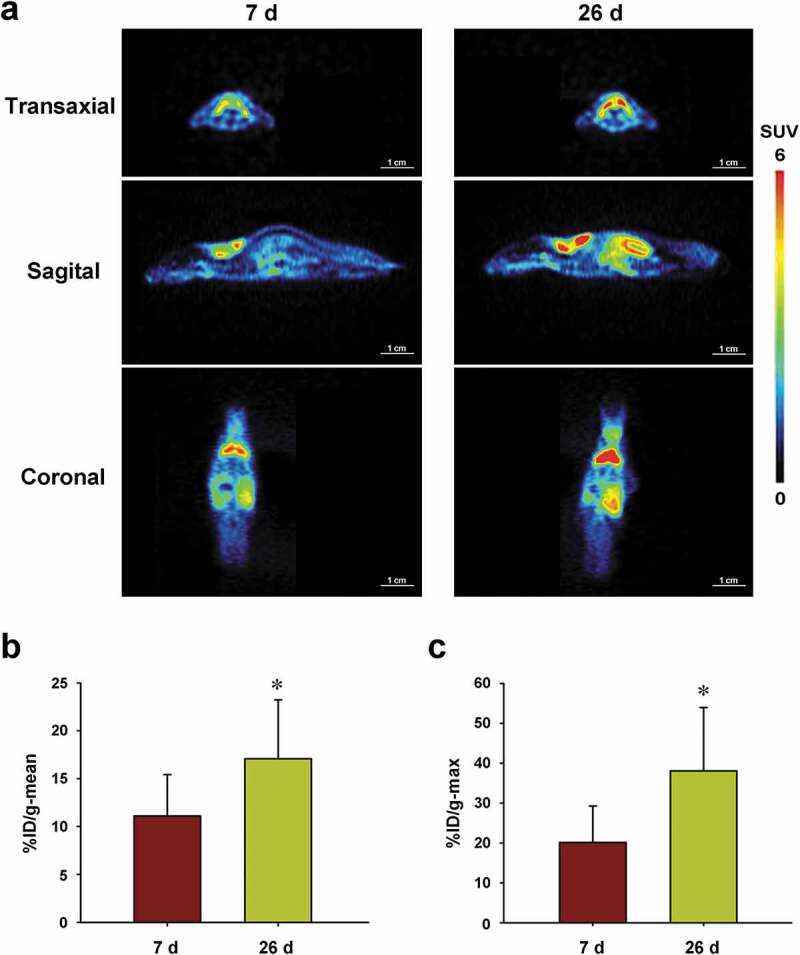
Time-longitudinal microPET imaging with [18F]DPA714 in CT26 xenograft mice

Furthermore, we conducted a time-longitudinal PET imaging study with [^18^F]DPA714 tracer in CT26 xenograft mice. We performed PET scans at day 7 and day 26 after tumour cell injection by [^18^F]DPA714 tracing. Similar to the previous findings, %ID/g-mean and %ID/g-max of the radioactive uptake of [^18^F]DPA714 in the scapular region were both the highest among all the organs (or ROIs) (as intuitively shown in Figure 4(a)). The %ID/g-mean (day 7, %ID/g-mean = 11.11 ± 4.32, n = 6; day 26, %ID/g-mean = 17.09 ± 6.15, n = 6) and %ID/g-max (day 7, %ID/g-max = 20.14 ± 9.14, n = 6; day 26, %ID/g-max = 38.07 ± 15.90, n = 6) of the radioactive uptake of [^18^F]DPA714 in the scapular region at day 26 were both significantly higher than those at day 7 (%ID/g-mean, p = 0.041; %ID/g-max, p = 0.026; day 26 vs. day 7) (Figure 4(b,c)). For cachexia, the parameters we observed include slow weight gain, weight loss, tissue necrosis, and depression. We observed that a small number of CT26 model mice (3 out of 13) had cachexia features such as slow weight gain, weight loss and tail necrosis, and died 10 days or more after the tumour cell transplantation, and were eliminated from subsequent study. Most of the tumour model mice did not have obvious cachexia characteristics. Body weight data of mice for the 26th day scans are shown in S Table 2. There were no significant differences in the body weights between different test time points.

PET scans were performed at day 7 and day 26 following tumour cell injection with radioactive tracer [^18^F]DPA714. (a) The radioactive uptake of [^18^F]DPA714 in the scapular BAT region was the highest among all the organs. (b) The %ID/g-mean and (c) %ID/g-max of the radioactive uptakes in the scapular region at day 26 were both significantly higher than those at day 7 (*p* = 0.041; *p* = 0.026; *n* = 6 mice for each time point).

## Discussion

In this study, we firstly evaluated the imaging efficacy of [^18^F]DPA714 for the monitoring of BAT in the CT26 colorectal cancer model, compared with the commonly used PET tracer [^18^F]FDG. As a specific ligand of TSPO protein, [^18^F]DPA714 showed a more stable and sensitive imaging of the BAT (which is rich in mitochondria and TSPO) than [^18^F]FDG in CT26 xenograft mice. Then, [^18^F]DPA714 PET also showed good quality imaging for BAT in the A549 and LLC tumour models. Furthermore, with the development of tumour, uptake of [^18^F]DPA714 by BAT in the late stage was significantly higher than that in early stage.

[^18^F]FDG PET imaging is the gold standard for the study of BAT activity in vivo [11], but its defects such as low positive rate (<5%) and poor stability in even normal physiological conditions limit the development of BAT research [12,13]. BAT mainly relies on fatty acids instead of glucose as the substrate of energy metabolism, so the uptake of [^18^F]FDG in BAT is relatively little. The imaging results of [^18^F]FDG PET depend on the imaging conditions and the stimulated activation level of BAT. The BAT [^18^F]FDG uptake can not only be affected by fasting status, diabetes, muscle activity and drugs (such as β-receptor blockers), but also related to the population characteristics (such as age, gender, and weight), temperature and seasons [11]. In addition, there are still some difficulties in the image quantification and analysis standardization for BAT imaging.

A large number of fat droplets and high concentration of mitochondria are typical characteristics of BAT. At present, most of the researches on BAT are focused on uncoupling proteins 1 (UCP1), expressed on mitochondrial inner membrane [24]. In fact, many proteins, including TSPO (expressed on the outer mitochondrial membrane), are also potential targets of BAT imaging. In contrast, WAT shows less mitochondria number and TSPO expression than BAT (including unstimulated BAT and beige fat) [25]. Recent studies have confirmed that TSPO specific tracers, such as ^11^C-PBR28 and [^18^F]FEPPA, can be used for the imaging of BAT and to detect the browning of WAT [19,25]. However, to the best of our knowledge, there is no published report about studying BAT activity with [^18^F]DPA714, a mitochondrial TSPO-based imaging tracer.

Nowadays, most of the studies on BAT have paid their attention to energy expenditure, obesity, insulin resistance and other energy metabolic disorders [6,26,27]. Scholars now have been trying to treat or ameliorate the energy metabolism diseases by promoting the browning of WAT. However, the browning of WAT may also accelerate the progression of cachexia in tumour patients [7,8,28,29].

Because tumour growth is not controlled or regulated by the normal physiological functions or processes, energy metabolism of the whole body is affected by the metabolic changes and challenges of tumour and the accompanied physiological stresses directly. As a key component in energy metabolism, BAT activity is also affected by tumour [28–30]. In this study, we observed that the uptake of [^18^F]DPA714 in BAT in the late stage of tumour was significantly higher than that in the early stage in the same tumour model. In patients with malignant tumours, the long-term presence of tumour tissue will cause chronic inflammation of the body. Inflammatory factors such as IL-6 and PTHrP, secreted by tumour tissues and mainly released by stimulating sympathetic nervous system and norepinephrine, act on β3-adrenergic receptor in adipose tissues and further activate the expression of related transcription factors and thermogenes such as UCP1, and induce the browning of WAT [7]. And this is not only an early event of tumour cachexia, but also runs throughout cachexia.

In conclusion, [^18^F]DPA714 is an ideal molecular imaging tracer to study the effect of tumour on the BAT activity and WAT browning. The safety of this tracer has been confirmed in several clinical subjects, and we also studied and confirmed that [^18^F]DPA714 can be used as a PET imaging tool for early warning and intervention efficacy evaluation of cachexia. We will further research the role of BAT in different tumour progression with this PET imaging tracer in the future preclinical and clinical studies.

## Supplementary Material

Supplemental Material
