# Supplementary figures and images for: Comparative [18F]FDG and [18F]DPA714 PET imaging and time-dependent changes of brown adipose tissue in tumor-bearing mice
Source: Adipocyte. 2020 Sep 9;9(1):542–9. doi: 10.1080/21623945.2020.1814546 (PMC7714432; doi:10.1080/21623945.2020.1814546)

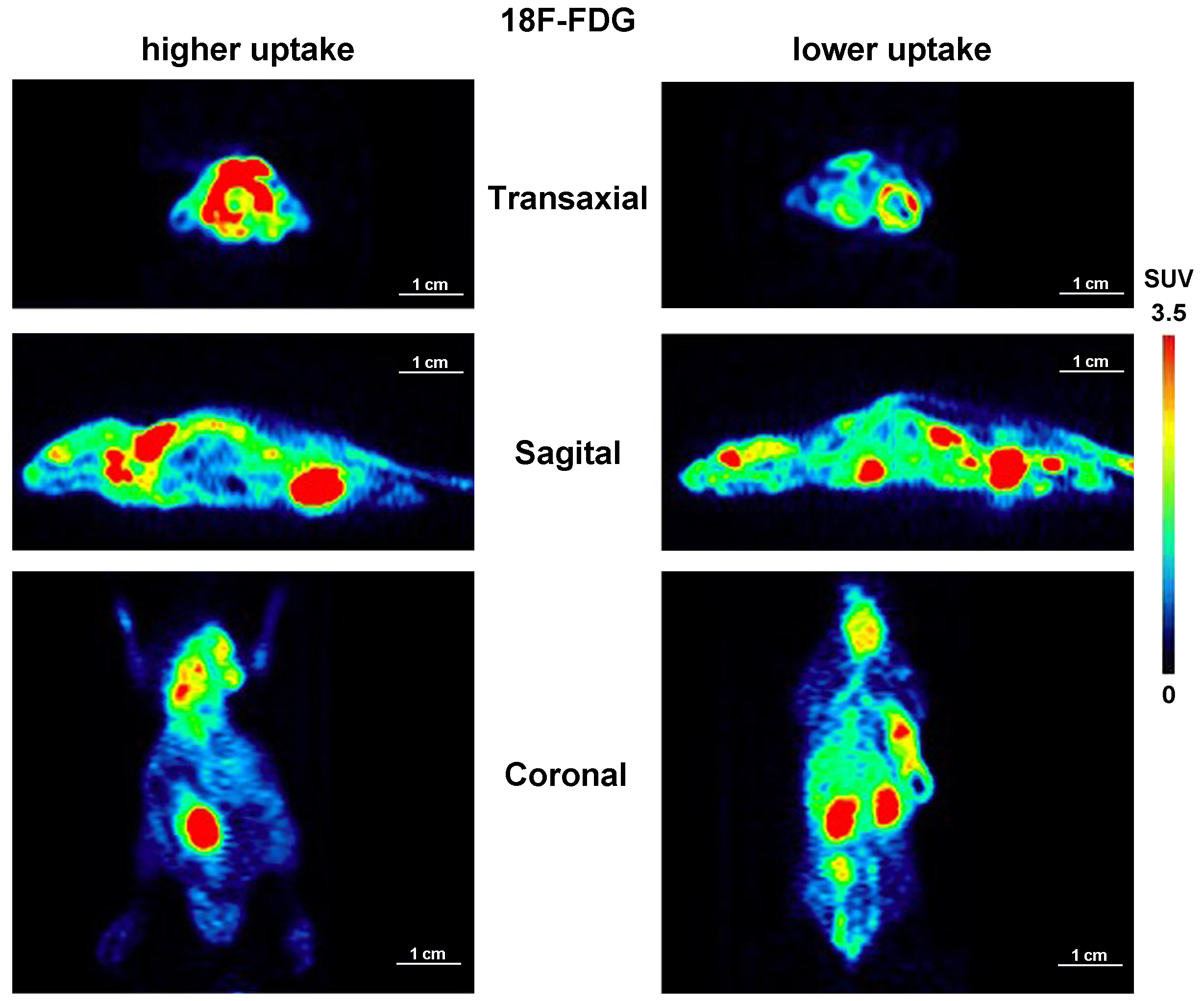

Supplement: Supplemental Material [file KADI_A_1814546_SM6943.tif]
